# “The Reynolds–McElhenney effect?”: effect of celebrity endorsement on colonoscopy-related videos on TikTok

**DOI:** 10.1016/j.igie.2023.01.005

**Published:** 2023-02-24

**Authors:** Ali Lahooti, Brian Critelli, Amier Hassan, Donevan Westerveld, Lindsay Rodgers, Maryam Gazi, Enad Dawod, SriHari Mahadev, Sonal Kumar, Carolyn Newberry, Reem Z. Sharaiha

**Affiliations:** Department of Gastroenterology & Hepatology, Weill Cornell Medical College, New York, New York, USA

## Abstract

**Background and Aims:**

Cases of young-onset colorectal cancer (CRC) have risen substantially over the last 20 years, and physicians are increasingly harnessing the influence of celebrities to raise awareness for CRC screening. Celebrity actors Ryan Reynolds and Rob McElhenney filmed their experiences undergoing colonoscopy as part of an initiative to raise awareness for CRC screening. Herein, we assess the quality of information on TikTok regarding colonoscopies and evaluate the effect of this celebrity endorsement.

**Methods:**

Hashtags encompassing colonoscopy were searched using TikTok’s algorithm, and 75 videos were considered. The DISCERN questionnaire and analytics were used to quantify the quality and popularity of each video.

**Results:**

Colonoscopy-related videos were higher in quality (*P* < .01) and popularity (*P* < .05) after the celebrity Reynolds–McElhenney endorsement compared with those released before. After the endorsement, the difference between the quality of the content created by physicians and nonphysicians was eliminated.

**Conclusions:**

Using TikTok, celebrities can aid in disseminating high-quality information regarding colonoscopy and other preventative medicine practices to young adults within the new social media landscape.

Colorectal cancer (CRC) is the second deadliest malignancy in the United States, accounting for over 50,000 deaths each year.^1^ Alarmingly, young-onset CRC cases have increased by over 50% since 1994, and adults born around 1990 have quadruple the risk of developing CRC compared with those born in the 1950s.^2^

Colonoscopy has been shown to decrease CRC mortality by detection and removal of precancerous adenomas and early detection of invasive carcinomas. A recently published randomized controlled trial demonstrated—for the first time—a reduction in CRC risk at 10 years for those who underwent screening colonoscopy compared with those who did not.^3^ Notwithstanding this landmark study, the estimated national colonoscopy compliance rate remains below the goal, and physicians are increasingly harnessing the influence of celebrities to raise awareness for CRC screening among young adults.^4^ For example, the advocacy of anchorwoman Katie Couric temporarily increased national colonoscopy screening rates in 2000.^5^ More recently, celebrity actors Ryan Reynolds and Rob McElhenney filmed their experiences undergoing colonoscopy to raise awareness for CRC screening.^6^

Despite similarities between this campaign and that of Couric, the social media landscape has dramatically changed over the last 20 years, and young adults have turned to newer platforms such as TikTok, the popular video-sharing platform, for education on health-related topics.^7^ Given the increasing incidence of CRC and the concurrent popularity of TikTok within this age cohort, we assessed the quality and popularity of colonoscopy-related videos on the platform, focusing on the effect of recent celebrity endorsement. We proposed, consistent with the “Couric effect,” that the Reynolds–McElhenney endorsement (RME) will increase the popularity of colonoscopy-related content.

## Methods

**Figure 1:**
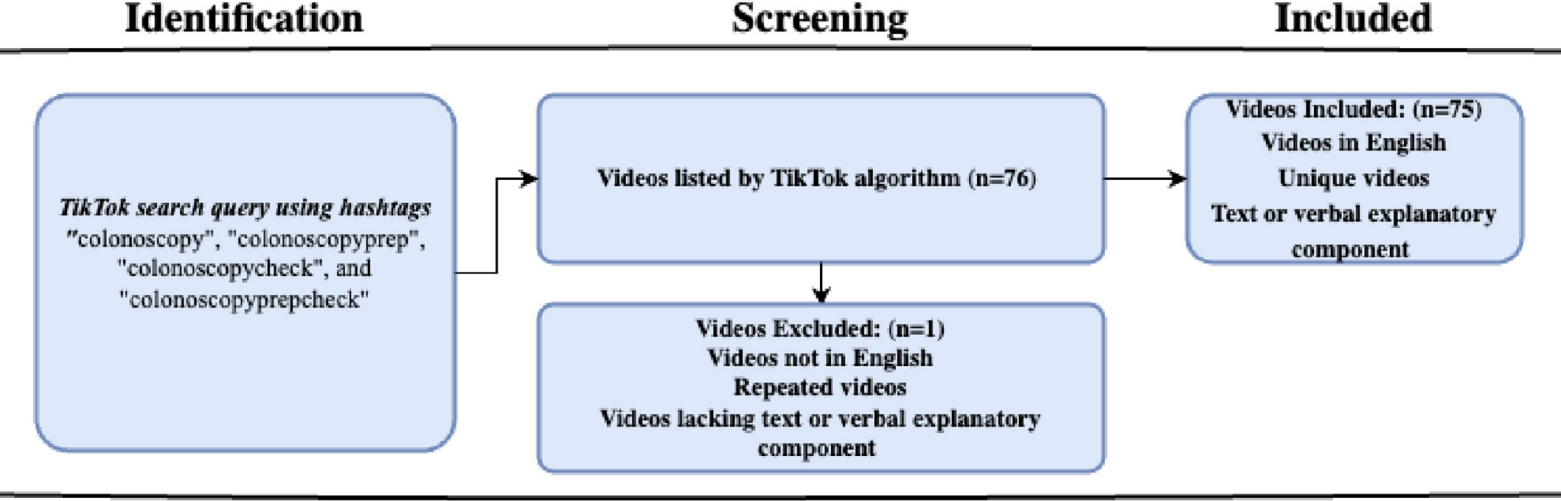
Study identification and selection overview. Videos were viewed based on TikTok’s hashtag search result algorithm.

Herein, our group used the same methodology as that in a previous study assessing weight loss procedure content on TikTok.^8^ We entered the hashtags “colonoscopy,” “colonoscopyprep,” “colonoscopycheck,” and “colonoscopyprepcheck” into TikTok’s search algorithm (Fig. 1). Videos were classified as “pre-RME” if they were published before the celebrity endorsement and as “post-RME” if published thereafter. The first 75 videos meeting inclusion criteria were considered.

Duplicate videos, non–English language videos, and videos without audio or visual explanation were excluded. For each video, descriptive statistics including content creator, views, likes, shares, uploader’s gender, publication date, and length were recorded. We used our method of visual categorization and stratified videos into 3 groups depending on the quality of supplementary visual aids. Two independent reviewers analyzed video content and descriptions to categorize and score each video using DISCERN, a validated tool assessing the quality of consumer health information. The DISCERN questionnaire consists of 16 questions on a continuous rating scale with a higher cumulative score correlating with higher quality and reliability of the assessed source. The grade of each video was averaged between the 2 reviewers.

Quality scores and popularity (views per day) were compared between physician-created videos and nonphysician-created videos and between videos published pre-RME and post-RME. Cohen’s kappa coefficient was used to assess the inter-rater reliability. The 1-way analysis of variance; 2-sample *t* test, assuming both equal and unequal variances where appropriate; and the Pearson χ^2^ test were used to determine statistically significant groups.

## Results

Of the 75 videos included in our study, 26.7% were created by physicians and 73.3% by nonphysicians (*P* < .001). The average DISCERN score for physician-created content was significantly higher than that of nonphysicians (*P* < .01), despite no significant difference in popularity (Table 1). Pre-RME, the top 10 most-popular videos had no significant difference in DISCERN scores compared with the 10 least-popular videos. Conversely, the top 10 most-popular videos post-RME scored significantly higher on DISCERN’s quality metric than the 10 most-popular pre-RME videos (*P* < .05) (Table 2). Across both time points, educational videos had significantly higher DISCERN scores than personal experience videos (*P* < .001). There was no significant difference in content creator or content category when comparing pre- and post-RME videos. Overall, the post-RME videos had significantly higher mean popularity (*P* < .05) and higher mean DISCERN scores (*P* < .01) than the content released pre-RME. There was an inter-rater reliability score >.8.

**Table 1:** Overview of popularity and quality of content on TikTok based on content creator profession and content type for colonoscopy videos

		Female gender n (%)	No. (%) of videos	Mean visual category	Mean no. of likes	Mean no. of shares	Mean time of video (s)	Mean DISCERN score	Popularity (views per day)
Content creator									
	Physician	7.0 (35.0)∗	20.0 (26.7)†	2.00	39,242.6	1861.4	56.2	31.1∗	7745.5
	Nonphysician	39.0 (70.9)	55.0 (73.3)	1.89	108,778.3	4615.4	49.9	25.1	12,983.6
Content type									
	Education	15.0 (53.6)	28.0 (37.3)‡	2.18∗	45,010.0	1819.1	65.7	34.2†	9665.4
	Personal experience	31.0 (66.0)	47.0 (62.7)	1.77	117,178.2	5109.3	43.1	22.3	12,731.5

∗ *P* < .01.

† *P* < .001.

‡ *P* < .05.

**Table 2:** Overview of popularity and quality of content on TikTok based on release date relative to the celebrity video release date for colonoscopy videos

	Female gender n (%)	Content creator (physician) n (%)	Content category (education) n (%)	No. (%) of videos	Mean visual category	Mean no. of likes	Mean no. of shares	Mean time of video (s)	Mean DISCERN score	Popularity (views per day)
Pre-RME	28.0 (60.9)	12.0 (26.1)	12.0 (26.1)	46.0 (61.3)	1.89	123,582.0	5540.80	43.7	24.5∗	6554.2†
Post-RME	18.0 (62.1)	8.0 (27.6)	13.0 (44.8)	29.0 (38.9)	1.97	37,340.7	1248.10	64.1	30.2	19569.5

*RME*, Reynolds–McElhenney endorsement.

∗ *P* < .01.

† *P* < .05.

## Discussion

Most adult TikTok users—over 80%—are between 18 and 44 years old, which coincides with the rising rate of early-onset CRC seen in this demographic.^2^^,^^9^ Thus, assessment of colonoscopy-related information on the platform is essential given the previously established relationship between media-based advocacy and changes in prospective patient interest.^5^ Our results suggest a relationship between celebrity endorsement and increased popularity of colonoscopy-based videos on TikTok. Not only were post-RME videos significantly more popular than pre-RME videos, but they were also higher in quality. At the pre-RME time point, the most- and least-popular videos did not differ in quality. However, post-RME, videos of higher quality were significantly more popular, which suggests that celebrity endorsements can contribute to the dissemination of more accurate information. This key finding, which establishes a postendorsement link between quality and popularity, could play a role in both popularizing and improving content in other areas of medicine.

Furthermore, after the endorsement, the observed difference between the quality of physician- and nonphysician-created content was eliminated, suggesting that the RME campaign reached the general public. Unsurprisingly, the quality of physician-created content was consistent pre- and post-RME, although this content experienced an increase in popularity after the endorsement. Previous studies analyzing social media content related to colonoscopy have shown that videos created by healthcare professionals are of higher accuracy and quality but of lesser prevalence.^10^ Collectively, our findings suggest that the RME correlates with an overall increase in popularity of colonoscopy-based content on TikTok and that such a campaign may improve the quality of content from nonphysician creators.

Limitations of our study relate to generalizability because of the use of relatively few hashtags, small sample size, exclusion of non–English language videos, and inability to predict changes to TikTok’s proprietary search algorithm. However, given prior analysis of the Couric effect and the results of our study, we suspect that high-profile celebrities can play a critical role in promoting preventive medicine practices to young adults within the new social media landscape.

## DISCLOSURE

*All authors disclosed no financial relationships.*
